# A systematic review of microsimulation models for skin cancer

**DOI:** 10.1186/s12911-025-03074-9

**Published:** 2025-07-01

**Authors:** Caroline G. Watts, Kirstie G. McLoughlin, Stephen Wade, Amelia K. Smit, H. Peter Soyer, Pablo Fernandez-Peñas, David C. Whiteman, Pascale Guitera, Gillian Reyes-Marcelino, Karen Canfell, Anne E. Cust, Michael Caruana

**Affiliations:** 1 The Daffodil Centre, The University of Sydney, a joint venture with Cancer Council NSW, 1 King St, Newtown, Sydney, NSW 2042 Australia; 2 Melanoma Institute Australia, The University of Sydney, Sydney, NSW Australia; 3 Frazer Institute, Dermatology Research Centre, The University of Queensland, Brisbane, QLD Australia; 4 Dermatology Department, Princess Alexandra Hospital, Brisbane, QLD Australia; 5 Faculty of Medicine and Health, The University of Sydney, Westmead Clinical School, Westmead, NSW Australia; 6 Department of Dermatology, Westmead Hospital, Westmead, NSW Australia; 7 Department of Population Health, QIMR Berghofer Medical Research Institute, Herston, QLD Australia; 8 Faculty of Medicine, The University of Queensland, Queensland, Australia; 9 Faculty of Medicine and Health, The University of Sydney, Sydney, NSW Australia; 10 Sydney Melanoma Diagnostic Centre, Royal Prince Alfred Hospital, Camperdown, NSW Australia

**Keywords:** Melanoma, Skin cancer, Microsimulation, Health decision modelling

## Abstract

**Background:**

Simulation modelling can assist with health care decision making. To inform the development and improvement of skin cancer focussed microsimulation models, we conducted a systematic review and narrative synthesis of published skin cancer models to assess the structure, parameterisation, and assumptions.

**Methods:**

The electronic databases OVIDMedline including Embase and the Cost-Effectiveness Analysis (CEA) Registry were searched up to 7 May 2025. Studies that included microsimulation of individuals who developed or had skin cancer were eligible for inclusion. No restrictions on publication date or language were applied. The outcomes of interest were the purpose of the models, characteristics of the models and applicability for modelling skin cancer screening.

**Results:**

Twenty-two models were identified from the systematic review. Nineteen papers modelled melanoma, and two papers modelled keratinocyte or non-melanoma skin cancer, and one paper modelled both melanoma and keratinocyte cancer. The models were developed to assess treatment strategies (*n* = 10), skin cancer screening programs (*n* = 7), diagnostic techniques (*n* = 3), post-diagnosis surveillance (*n* = 3), preventative interventions (*n* = 1) and time to treatment (*n* = 1), with three models reporting dual aims. There was substantial variation in the simulation of the natural history of melanoma between models, with more recent models having separate natural history and clinical modules. The quality was assessed using the Quality Assessment Reporting for Microsimulation Models (QARMM) checklist and the majority of models were assessed to be of moderate quality. Limitations from these models included assuming an average tumour behaviour and constant melanoma development and progression over time. Data availability was also noted as a limitation for some models.

**Conclusions:**

Most microsimulation models related to skin cancer have focused on late-stage treatment strategies. Tumour characteristics, apart from stage at diagnosis, were not accounted for in most models.

**PROSPERO registration number:**

CRD42024504250.

**Supplementary Information:**

The online version contains supplementary material available at 10.1186/s12911-025-03074-9.

## Introduction

Around 1.2 million keratinocyte (non-melanoma) skin cancers and over 330,000 melanoma skin cancers are estimated to occur globally each year, and the incidence of skin cancer is increasing [1]. Australia and New Zealand have the highest rates of melanoma and keratinocyte skin cancer in the world [1] and it is estimated that two in three Australians will be diagnosed with skin cancer during their lifetime [2]. For melanoma, which is responsible for the majority of skin cancer deaths, there is an association between early detection and lower morbidity and mortality [3].The most important cause of skin cancer is ultraviolet (UV) radiation, although genetic and behavioural factors also contribute to risk [4–7]. Skin cancer prevention campaigns in some countries have been effective at reducing UV exposure and increasing public awareness of skin cancer risks [5, 6, 8]. Early detection strategies to reduce morbidity and mortality are also required to reduce the impact of skin cancer [9, 10]. Observational studies have indicated a decrease in mortality risk of melanoma is associated with routine skin checks [11] or having a recent skin examination in the past three years [12], but large randomised controlled trials have not been conducted. Costs associated with skin cancer management, including treatment of later stage disease, have been increasing over time [13, 14] and these costs alongside inequities in access and variable quality of care has led to consideration of organised screening as a potentially cost-effective and more equitable model of early detection compared to opportunistic screening in high-incidence countries like Australia. However, more evidence is required about the potential benefits, harms and costs of screening [15]. Simulation modelling can assist with health care decision making and policy such as whether to introduce an organised screening program, as different scenarios including the type of screening test, management pathways and eligibility criteria can be modelled and the disease burden and costs and benefits evaluated accordingly [16, 17].

A systematic review of cancer screening models [17] published between 1999 and 2018 including for breast, lung, colorectal, prostate, and cervical cancers found individual-level models (86 studies) were marginally more widely used than cohort level models (84 studies) to simulate cancer interventions, however this masks the declining use of cohort level and increasing use of microsimulation approaches since 2011 [17].The advantage of microsimulation models over cohort models is that they can simulate events and lifetimes from the perspective of the individual, and variation seen in small groups such as family units or subpopulations [16]. These simulations can include heterogenous aspects of individual characteristics, such as health components like cancer risk, behaviours and effects of interactions [16].

Cancer microsimulation models have been used to model costs and benefits of alternative interventions to guide vaccination (e.g. cervical cancer) [18–20] and screening (e.g. bowel and lung cancer) [21–23] policy decisions. In Australia, we are developing a microsimulation model for melanoma (“Policy1-Melanoma”) to examine the costs, benefits and harms of a range of competing skin cancer screening policy scenarios. The Policy-1 platform (see Supplementary file) comprises a suite of cancer control models and modelling tools designed to simulate and evaluate cancer prevention and control interventions across diverse settings, including high-income, low-income, and lower-middle-income countries [18–22 [24, 25],. To inform the development of this model, we conducted a systematic review and narrative synthesis of published skin cancer microsimulation models to assess their structure, parameterisation, and assumptions.

## Materials and methods

### Search strategy and selection criteria

We aimed to include skin cancer microsimulation models that examined the population level burden of skin cancer from an individual perspective, and to assess their strengths and weaknesses. The search was registered with the international prospective register of systematic reviews (PROSPERO, ID#CRD42024504250). A search was conducted on OVIDMedline, which included the databases Medline and Embase using the MeSH terms “melanoma*” or “skin cancer*” with “microsim*” or “agent-based model*” or “individual-based model*”, and the Cost-Effectiveness Analysis (CEA) Registry [26] using the terms “melanoma” or “skin cancer”. The search covered the period from 1 January 1987 up to 7 May 2025 (see search strategy in Supplementary materials). Studies were included if they included people with skin cancer or who developed skin cancer and were modelled from an individual perspective.

Using Covidence [27], CW and KM independently screened all articles for relevance and consensus was reached through discussion with the review team (AEC, AKS and MC). We only included models that explicitly simulated individuals as these were the most relevant to inform purposes of the review. Models that simulated ‘average’ members of a representative cohort or sub-groups, or that specifically focused on oncogenesis or tumour growth at the cellular level were excluded. We included both discrete-time and discrete-event simulations. Full text reviews were restricted to English. If the model had been adapted from a previously published model, then the original model was sourced using the reference list and included in the review.

### Main outcomes

The main outcomes assessed were descriptions of the purpose and characteristics of the models, and applicability for the models for evaluating skin cancer screening.

### Data extraction

A data extraction template was designed using Excel. Data extraction was conducted by CW and KM and cross-checked by AKS, and any discrepancies were resolved through discussion with AEC and MC. Data items extracted included author, year of publication, country, population modelled, model purpose, simulation method, data sources used, model states described, model assumptions, and applicability to skin cancer screening. In reporting the review, we followed the Preferred Reporting Items for Systematic reviews and Meta-Analyses (PRISMA) statement 2020 [28]. A narrative synthesis was undertaken to summarise the strengths and limitations of models in the included articles.

### Quality assessment

CW and KM assessed the quality of the microsimulation models using the Quality Assessment Reporting for Microsimulation Models (QARMM) checklist [29, 30]. The model purpose, data, transparency, uncertainty, validation and generalisability were assessed using a 15-point check list with each item scoring 0, 0.5 or 1 point depending on completeness. Disagreements were discussed and resolved. Based on a published scoring algorithm [30], models that scored 75% or greater (11.5/15) were considered high quality models, less than 30% (5/15) low quality models, and 30–74% as moderate quality.

### Patient and public involvement

No patients or consumer representatives were involved in the design or conduct of the review.

## Results

### Search results

**Fig. 1:**
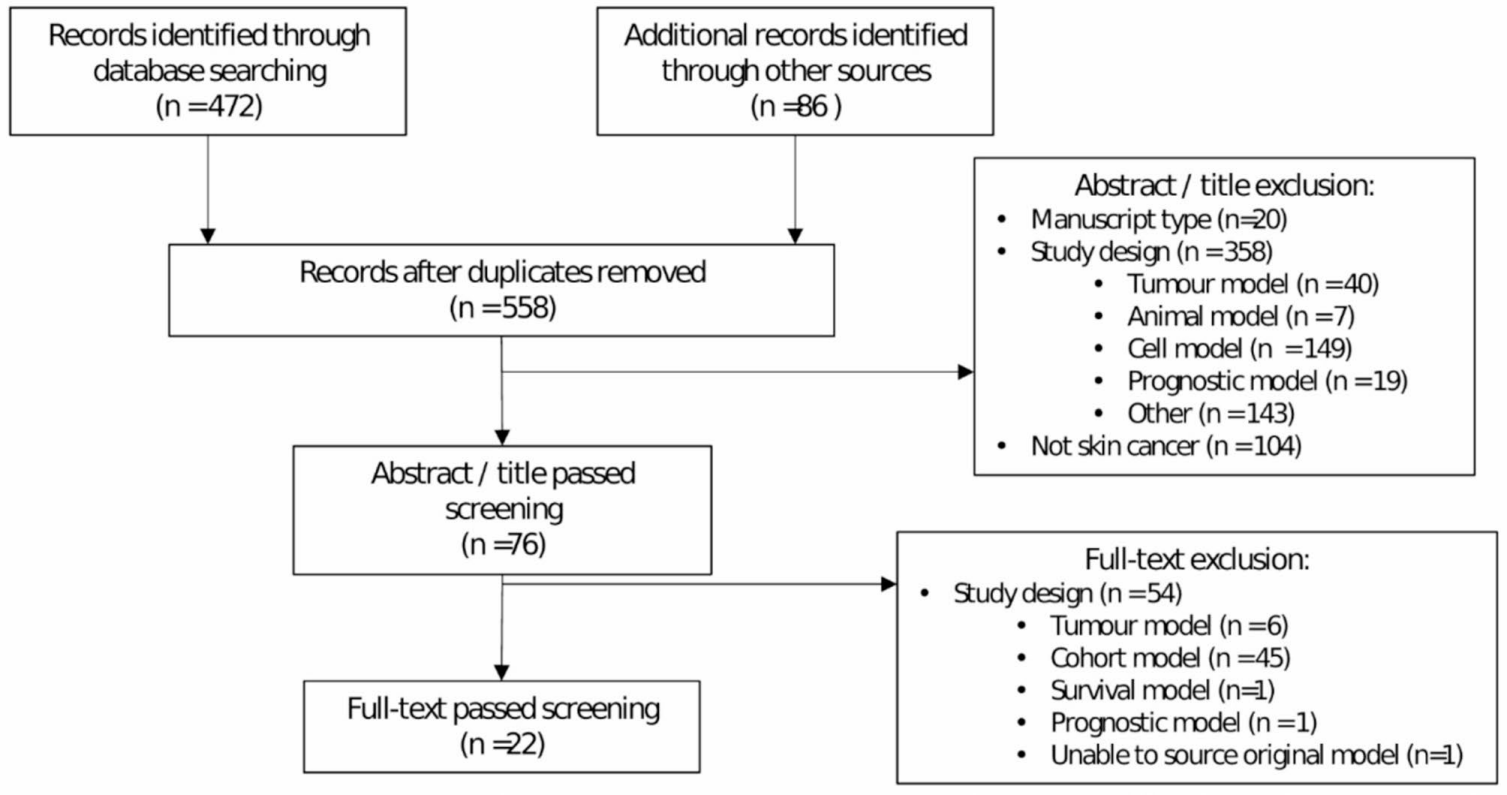
PRISMA diagram showing process of study selection. No additional articles were included from reference lists. Four papers referred to adapting earlier models which were included in the review

The search retrieved 558 abstracts; 472 through OVIDMedline and 86 from the Cost-Effectiveness Analysis (CEA) Registry (Fig. 1). Following review of 76 full text papers, 22 papers were retained for data extraction.

Just over half of the papers reviewed (13/22) were published in the past six years (since 2018). The papers described models based on populations in the USA (11 papers [31–41]), Canada [42], Europe (nine papers, of which five in the UK [43–47], Sweden [48], The Netherlands [49], Germany, two papers [50, 51]), and Australia [52]. Eight papers modelled the general population [31–33, 35, 47, 48, 50, 51], while the remaining models were based on subsets of the population, mostly patients with melanoma (Table 1).

**Table 1:** Articles included from literature review with relevant data extracted

Author (Year)	Model aim	Population	Country	Data sources (trial name)	Simulation method: discrete time^2^ vs. time-to-event (and software)	Model states	Model application and comparators
***Preventative impact***							
*Eskander (2021) *[31]*.*	Prevention impact.	General population. Starting age 14–17 years	USA	US Centers for Disease Control and Prevention melanoma data, published literature.	Discrete time (1-year). (TreeAge^4^).	Well, melanoma diagnosis [stages I-IV], death (MSM & OCM).	CEA of tanning bed bans compared to no ban.
***Screening & early detection***							
*Eisemann (2015) *[50]*.*	**Screening strategies.**	General population, starting age 20 years	Germany	Cancer registry (Association of Population-Based Cancer Registries in Germany: GEKID).	Discrete time. (not described)	Well, melanoma, melanoma diagnosed, death (MSM & OCM).	To examine the Impact of screening program on melanoma incidence & mortality rates. from 35 years (1, 2 & 3-year intervals).
*Gomez Rossi (2022) *[33].	Diagnosis strategies.	General population.	USA	Published literature.	Discrete time (1-year) (TreeAge).	Well, melanoma, accurate melanoma diagnosis [yes/no, early & late], recurrence [late], death (MSM, OCM).	CEA of AI supported diagnosis compared to standard dermatologist care.
*Losina (2007) *[35]*.*	**Screening strategies.**	General population. Family history (high & low risk).	USA	Cancer registry (SEER), hospital-specific data, expert elicitation.	Discrete time. (not described)	Well, melanoma, melanoma diagnosis [true & false, stages I-IV], PFS, death.	Impact of screening (single or, 1, 2 & 5-year intervals) compared with background screening alone. Target population impact.
*Okafor (2013) *[41]* extends Malone *[57]	**Screening strategies**	People with Crohn’s Disease	USA	Published data	Discrete time (1-month). (TreeAge).	CD states, NMSC [local or distant], death (ACM, CD, BCC, SCC)	CEA of different strategies to screen for non-melanoma skin cancers in people diagnosed with Crohn’s disease.
*Walpole (2021) *[52]*.*	**Screening strategies.** Surveillance strategies.	BAP1 gene carriers.	Australia	Guidelines, published literature, trial data (CheckMate 067).	Discrete time (TreeAge).	Well, melanoma, melanoma diagnosed [early & late], death (MSM & OCM)	Impact of screening and surveillance (6-monthly interval).
*Wilson (2018) *[47]	**Screening strategies.**	General population.	UK	Cancer registry, cohort study, published literature, expert elicitation.	Discrete time (R software).	Well, melanoma [histology, stage], death	CEA of population based screening programs based on risk of melanoma using a melanoma risk assessment tool (Williams)
*Gogebakan (2020) *[32]*.* Extends *Birnbaum* [56]	**Population-based screening.** Treatment impact.	General population. High risk only.	USA	Cancer registry (SEER), published literature.	Discrete event (R software).	Well, melanoma diagnosis [early & late], death (MSM & OCM)	Impact of screening program (present & absent). Impact of adjuvant therapy.
Baltus [51] (Based on Eisemann)	**Screening strategies.**	General population.	Germany	Federal Statistical Office of Germany, Insurance data, Cancer registry (Association of Population-Based Cancer Registries in Germany: GEKID), Published literature	Discrete time (R software).	Well, melanoma, melanoma diagnosed, death (MSM & OCM, BCC, SCC).	To examine the costs and Impact of a range of screening programs for people (aged 35 years to 85 years over 30 years) on melanoma incidence & mortality rates. Programs compared included no screening, status quo (2-year) & 3-year intervals, and with increased participation.
***Surveillance & management options***							
*Claeson (2016) *[48]*.*	Population projections. Diagnosis strategies.	General population.	Sweden	Cancer registry, published literature, hospital data.	Unclear. (ithink)^1^.	Unclear.	Impact of screening program on melanoma incidence including change in time to diagnosis related to patient or doctor delay.
*Kontogiannis (2022) *[44]*.*	Surveillance strategies.	People with–Stage I melanoma.	UK	Hospital-specific data, cancer registry, systematic review, guidelines. published literature, expert elicitation.	Discrete time (1-month). (TreeAge).	Treatment, recurrence [stages I-IV], death (MSM & OCM).	To compare the impact of surveillance strategies by level of diagnostic expertise and screening interval (clinician type & 3, 4, 6, & 12-month intervals).
***Surveillance***,*** management & diagnosis options***							
*van der Meijde (2016) *[49]*.*	Surveillance and diagnosis strategies. Treatment options.	Melanoma patients.	The Netherlands	Cancer registry, guidelines, published literature, retrospective cohort, expert elicitation.	Time-to-event (C + + software).	First melanoma diagnosis [local, regional, distant], PFS, melanoma symptoms, recurrence, death (MSM & OCM). Clinical management overlays.	Model simulating the progression diagnosis and management of melanoma.
***Treatment options***							
*Almutairi (2019) *[39]	Treatment options.	Patients with stage IIIB, IIIC and IV melanoma	USA	Trial data	Time-to-event. (not described).	PFS, progression or death.	CEA of adjuvant therapy of single vs. combination therapies.
*Beale(2013)[45]*	Treatment options	Patients with BRAF mutant stage IIIC and IV melanoma	UK	Trial data, registry data, published literature	Time-to-event. (not described).	PFS, post-progression survival (PPS), and death.	CEA comparing two alternative adjuvant therapies.
*Crott (2004) *[42]*.* Extends *Hillner *[54]*.*	Treatment options.	Patients with stage II and III melanoma.	Canada	Trial data (ECOG E1684), published literature.	Discrete time (1-year). (@Risk Software^3^; Excel)	Treatment, PFS [continuing treatment & off treatment], relapse, death (MSM).	Trial replication. CUA of adjuvant therapy (high-dose interferon) compared to watchful waiting over 7 years and lifetime time horizon.
*Gibson (2019) *[43]*.*	Model comparison^5^. Treatment options.	Patients with Stage III and IV melanoma	UK	Trial data (CheckMate 067).	Time-to-event. (Excel).	Treatment, PFS, relapse, death.	Model type comparisons. CEA of adjuvant therapy based on phase III trial for evaluation of single vs. combination therapies.
*Li (2023) *[34]*.*	Treatment options.	Patients with BRAF mutant-stage III and IV melanoma excised.	USA	Trial data (IMspire150, COMBI-AD, COLUMBUS).	Discrete time (28-days). (TreeAge).	Melanoma diagnosed, treatment [PFS, relapse], death (adverse events & OCM). Clinical management overlays.	CEA comparing a range of adjuvant therapy treatment options.
*Mojtahed (2021) *[36]*.*	Treatment options.	Patients with BRAF mutant-stage III melanoma excised.	USA	Published literature, trial data (unspecified).	Discrete time (6-weeks). (TreeAge).	PFS, recurrence [local or distant], death.	CEA comparing a range of adjuvant therapy treatment options.
*Kansal (2013) *[40]	Diagnosis strategies.	Patients with a biopsied suspected melanoma	USA	Registry data, published literature.	Hybrid design. Event and Discrete time (1-year) (Excel).	Excision, Provisional diagnosis (TP, FP, TN, FN), Melanoma diagnosis [stages I-IV], death (MSM &OCM)	CEA comparing two diagnostics strategies- both compared excisional biopsy based on microscopic assessment alone compared to assessment including the use of a FISH assay.
*Lee (2019) *[46]* extends Larkin *[55]	Model comparison, Treatment options.	Patients with BRAF mutant-stage III and IV melanoma.	UK	Trial data, Published data	Discrete time (Excel)	PFS, recurrence, death (MSM).	CEA of adjuvant therapy of single compared to combination therapies.
*Seidler (2009) *[37]*.*	Treatment options.	NMSC patients.	USA	Prospective cohort. Published literature.	Discrete time (TreeAge).	NMSC diagnosis, treatment, PFS, recurrence.	CEA of Mohs micrographic surgery compared to standard surgical excision.
*Tarhini (2018) *[38]*.*	Treatment options.	Patients with untreated unresectable stage III and IV melanoma. All BRAF mutations.	USA	Trial data (CheckMate 067 & 069, KEYNOTE-006), expert elicitation.	Time-to-event (Excel).	Treatment [first-, second- & third-line], PFS, death.	CEA comparing a range of adjuvant therapy treatment options.

Square brackets (‘[]’) indicate states which are competitive options within a bigger state ‘type’

ACM all-cause mortality; BCC basal cell carcinoma; CEA, cost-effectiveness analysis; CD Crohn’s disease; CUA, cost-utility analysis; FISH Fluorescence in situ hybridization; MSM, melanoma-specific mortality; OCM, other-cause mortality; PFS, progression-free survival; SCC squamous cell carcinoma; TP true positive; FP false positive; TN true negative; FN false negative

Published literature refers to where specified data were collected from a single specified publication. Proprietary software: https://www.iseesystems.com/, ^2^ Cycle length. This is only included where the model was discrete time, and the authors explicitly stated the time increments. ^3^ Proprietary software: https://lumivero.com/products/at-risk/^4^ Proprietary software: https://www.treeage.com/^5^ The model comparison within this paper includes a range of model implementations and structures. Only one of these models was an individual-based simulation, the characteristics included in this table therefore only relate to the microsimulation component

### Purpose of the models

Most of the papers (20/22) modelled melanoma diagnosis and/or treatment, and three papers [37, 41, 51] simulated the diagnosis or treatment of keratinocyte cancer. One paper [51]modelled the development of both melanoma and keratinocyte cancer, but a person could develop either a melanoma or a keratinocyte cancer only once. The application of the models varied. Of the melanoma models; 10 models were developed to compare the cost-effectiveness of adjuvant treatment options for advanced melanoma [32, 34, 36, 38, 39, 42, 43, 45, 46, 49]. Six models were developed to consider the effectiveness of skin cancer screening and/or surveillance pre-diagnosis [32, 35, 47, 50–52]. Four papers examined the effectiveness of surveillance [44, 49, 51, 52]. Two papers compared diagnostic techniques [33, 40]. One paper assessed the impact of a policy change intervention [31], and one assessed the impact of screening on time to diagnosis [48]. For keratinocyte cancer models, two papers compared screening strategies [41, 51] and the other treatment options [37] (Table 1). Fifteen studies examined cost-effectiveness and all used a healthcare payer perspective [31, 33, 34, 36–43, 45–47, 51].

Data sources were from cancer registries, research studies (including prospective and retrospective cohorts, and randomised clinical trials), clinical databases, systematic reviews, guidelines, and expert elicitation. Most models used combinations of cancer registry, clinical trial or cohort studies, and literature inputs. Eight models included trial data to examine the cost-effectiveness of adjuvant treatment options for advanced melanoma [34, 36, 38, 39, 42, 43, 45, 46].

### Characteristics of the models

Most models were discrete time simulations (15 models [31, 33–37, 40–42, 44, 46, 47, 50–52], developed using TreeAge (eight models [31, 33, 34, 36, 37, 41, 44, 52]), Excel (five models [38, 40, 42, 43, 53]) or other statistical software packages (six models [32, 42, 47–49, 51]). The software used was not described for four models [35, 39, 45, 50]. Five models [32, 41, 42, 46, 51] were developed from previously published models [50, 54–57] (Table 1).

Of the melanoma models, nine models [31–33, 35, 40, 47, 50–52] started from a ‘well’ state (i.e. no underlying melanoma), although three of these models started with a person presenting with a suspicious lesion that could be melanoma [40, 47, 51]. Four models started with a person with a confirmed melanoma diagnosis [32, 44, 48, 49], of which three models were based on individuals presenting with an invasive melanoma [32, 48, 49], and the other modelled individuals with a recently excised stage I melanoma [44]. Eight models focused on the management of late stage disease [34, 36, 38, 39, 42, 43, 45, 46] and of these models, seven commenced from Stage III melanoma, and one study modelled patients with Stage II or III melanoma [42].

Of the keratinocyte cancer models, two model simulated individuals prior to diagnosis [41, 51] and the other commenced with a patient presenting with a keratinocyte cancer [37].

Five papers incorporated inherited conditions that predispose individuals to an increased risk of skin cancer. One paper incorporated increased risk of keratinocyte cancer [41], and four incorporated increased risk of melanoma due to genetic factors [32, 41, 47, 52]. One paper incorporated an increased risk of melanoma due to sunbed use [31].

All the melanoma models contained transitions for probabilities of invasion derived from stage at diagnosis. Three models [47, 49, 51] developed frameworks with interacting, distinct, tumour growth and clinical modules.

### Applicability to skin cancer screening

Population based skin cancer screening models varied in the age skin cancer screening commenced, for example from 14 to 17 years [44] based on the use of sunbeds, 18 years [52], 20 years [50], 35 years-75 years [32], 35–85 years [51], 50 years [35] or not defined (general population) [47] for melanoma screening. The keratinocyte cancer screening model [41] was based on outcomes for people with Crohn’s disease and assumed an average age of 30 years as the starting point based on the mean age of diagnosis for Crohn’s disease. Some models also incorporated opportunistic presentation of a person for a skin check [47, 52]. Models looked at outcomes after 25 years [32], 30 years [47] and over a lifetime [35, 50–52].

Of the models examining skin cancer screening, most models [35, 41, 47, 50–52] incorporated the effect of changing screening intervals. Risk scores used in two models [32, 47] incorporated sex, age, hair colour at age 15, density of freckles on arms before age 20, history of severe sunburn in childhood and adolescence (2–18 years), number of raised moles on the arms, and history of keratinocyte cancer, and examined the effects of changing screening strategies between subgroups. One model incorporated level of risk based on personal and family history of melanoma [35]. One model varied the probability of participation in a screening program [51]. The model examining risk of keratinocyte cancer [41] also incorporated increased risk due to immune suppression following treatment initiation for Crohn’s disease.

In addition, seven models [33, 35, 47–51] included a pre-diagnosis or an explicit ‘undiagnosed melanoma’ state with the possibility of stage progression whilst undiagnosed. Six [33, 35, 47, 48, 50, 51] of these models were early detection interventions (Supplementary Table 1), and the other model simulated patient management from their first diagnosis [49]. None of the simulations distinguished melanoma diagnosed at routine skin checks from those brought to clinical attention on the appearance of a suspicious mole or spot. However, two papers (from the UK [47] and Sweden [48]) did model opportunistic skin checks and diagnosis of melanoma. Of these studies, one study included two pathways for a patient to present to their GP for a skin check, either of their own initiative with a suspicious lesion or because they had been advised to do so following an online risk assessment [47]. The other study modelled the effects of earlier diagnosis related to improved doctor diagnostic skills or increasing patient awareness [48]. Two models [47, 51], included diagnosis of in situ melanomas (stage 0 melanoma).

Among models incorporating the detection of melanoma or keratinocyte cancer, most included additional measures of diagnostic accuracy such as including false positive results [33, 35, 40, 41, 47, 51, 52]. Other models used strategies including registry-based stage at diagnosis data [31, 32, 48, 50, 51]. Four models incorporated overdiagnosis [32, 40, 50, 51].

Fifteen models simulated the clinical pathways of patients following a diagnosis of melanoma; of these eight modelled scenarios based on the general population [31–33, 35, 47, 48, 50, 51], and three incorporated risk-stratified screening [32, 41, 47]. One paper incorporated a subsequent diagnosis due to screening [52].

Tumour growth was explicitly simulated in four models [47, 49–51], which accounted for variability in melanoma growth rates that would be expected to change the utility of screening. Claeson [48] incorporated two melanoma growth rates (high and low) based on histological subtype, influencing when a melanoma could be detected. For the other models, tumour growth was incorporated by varied transition rates through explicit stages of disease or thickness categories. Models that compared adjuvant therapies incorporated high-risk melanomas and melanoma risk of recurrence predominantly based on trial data [34, 36, 38, 39, 42, 43, 45, 46]. One model also incorporated a toxicity score [49].

Similarly, for models simulating melanoma progression, there was variation in the incorporation of tumour recurrence or surveillance post diagnosis. One model explicitly examined surveillance following an excision of a Stage I melanoma [44]. In other models, surveillance was incorporated into the model and recurrence was based on stage at diagnosis [49]. Five models [44, 47, 49, 51, 52] were developed to assess surveillance strategies targeted to European populations (Table 2). As there are limited data available on melanoma progression and recurrence [58], these models used multiple, distinct datasets to infer likely transition probabilities (Table 1).

**Table 2:** Strengths, limitations, and notable assumptions of the models included in the review, with respect to their initial research purpose

Model	Model characteristics and assumptions	Strengths	Limitations^1^
***Preventative impact***			
Eskander 2021 [31]	• Two intervention scenarios: no sun-bed ban and complete sunbed ban. No follow-on changes from < 18 years ban (i.e. rates remain as seen in current population). • Assume stage distributions as in the current general population in both intervention arms. Increased risk associated with tanning beds is not linked to diagnostic stage [82]. • Mortality from any state (including recurrence state) is initial stage dependent.	• **Representative population** (adjusted to reflect population demographic characteristics). This accounts for potentially lower impacts associated with an aging population. • **Heterogeneous intervention impact**. The model uses a probability distribution to allocate tanning bed usage and accounts for age-variation in use [82]. • Both **progression and recurrence** simulated with probabilities dependent upon initial diagnostic state.	• **Melanoma rates taken directly from incidence**. This ignores any birth-cohort or period trends. • **No distinction between MSM and OCM in death outcomes**. Tanning bed use preferences may change OCM in addition to MSM or be linked to social-economic factors that mitigate MSM survival benefits. • No dose-variable impacts. The full increase in risk implemented for each tanning bed user independent of usage frequency. Usage is known to have an impact on melanoma risk [83]. • **No validation** of the model was performed [84].
***Screening and early detection***			
Eisemann 2015 [50]	• All undiagnosed melanoma is detectable (i.e. at least 0.01 mm). • Exponential tumour volume growth (doubles every 94 days). Age at tumour inception back-calculated from age at diagnosis, tumour volume, and rate of growth. • **No birth-cohort or period effects** simulated. This may not be a problem in Germany before 2008 when a skin cancer screening campaign was introduced [85]. • Screening only between 35 and 85 years old, with minimum screening interval of two years. People with a previous history of melanoma excluded. • No death from melanoma > 15 years post-diagnosis • No changes in mortality over time. No increase in mortality observed in most countries despite increasing incidence. Excludes impacts of novel treatments.	• **Representative population**: simulated cohort transformed to reflect observed demographic structures. • Separate simulations by sex. This reflects biological differences between the sexes in life expectancy and melanoma incidence/prognosis. • Variable tumour characteristics (thickness and growth rates). Tumour thickness at diagnosis depends on age: in accordance with observed data [86]. Tumour growth rates are drawn from a distribution (most slow, some fast): this approximates the growth variations associated with histological subtype [87–89]. • **Overdiagnosis estimates** incorporated. • Tumour (thickness) and patient (age) characteristics affect survival [90]. • Varied scenarios. Several screening strategies simulated with distinct participation (20%), test sensitivity (80%) based on German diagnostic expertise), and intervals (2-years vs. 3-years). • Calibrated against registry-derived stage and observed age-standardised incidence for 2007 only. • Sensitivity analyses for tumour growth rates and screening intervals. Variability assessed in terms of both input distributions and draws.	• **No melanoma in situ** diagnosed. • **Single lifetime melanoma event**. A first primary melanoma is a high-risk factor for subsequent events [91]. A single melanoma event may underestimate the overall benefits and harms of the intervention. **No recurrence** (progression) possible. • **Undiagnosed melanoma rates taken directly from incidence**. This is adjusted to be approximately 10 times the observed incidence. • **All diagnoses accurate**. No misdiagnosis, false negatives, or false positives. This is inconsistent with clinical diagnosis of melanoma [84, 92]. This may underestimate the harms [93] and overestimate the benefits associated with the intervention. • No non-screen-related **incidence trends**. Incidence has increase in many countries [77, 78] without screening programs. Excluding these trends is inconsistent with melanoma epidemiology [84] and underestimates the benefits associated with the intervention. • **No self-detection**. Likelihood of **detection by doctor does not differ** by melanoma subtype or growth rate. • **Survival is independent** of some melanoma criteria - AJCC staging categories N (extent of spread to the lymph nodes ) or M (presence of metastasis )status [94]. Survival without screening will be notably higher than expected. This underestimates the intervention impact. • **No validation**. Comparison to stage at diagnosis but no comparison to observed histological subtype proportions/growth variations. No comparison to observed mortality [84].
Gomez Rossi 2022 [33]	• Dermatologist screening program for individuals age 50 + years. • Simulation starts at age 48. • Using a dermatoscope with and without artificial intelligence (AI).	• Parameters were selected from a distribution of sensitivities and specificities were used	• **No melanoma in situ** diagnoses. • Melanoma development and stage are **age independent**. This is inconsistent with the epidemiological characteristics of melanoma [84]. • **Fixed undiagnosed progression** rate. This does not reflect known melanoma biology [84]. • **All diagnoses accurate**. **No overdiagnosis** incorporated. **No self-detection included** and the likelihood of **detection by doctor does not differ** by any variables. • Recurrence risk is stage independent and explicitly only advanced stage. • **No validation** of the model was performed.
Losina 2007 [35]		• **Varied diagnostic accuracy**. Diagnoses could be false negatives. • Incorporates **routine skin checks**. • **Stage-dependent** (local, regional, distant) progression, recurrence, and survival. If local disease **thickness-dependent** too. • **Sensitivity analyses** for stage-survival, incidence, prevalence, progression (2–36%), increased risk in siblings, screening. PSA for costs, specificity, sensitivity, and progression rate.	• **No melanoma in situ** diagnoses. • **Single lifetime melanoma event**. No second primary diagnoses or recurrence. A single melanoma event may underestimate the overall benefits and harms of the intervention. • **Fixed age-dependent** incidence and prevalence rates. • **Fixed stage progression** (10% tumours yearly). • **No incidental or patient detection**. Diagnosis possible at routine skin check or screen event only. All **individuals comply completely** with routine skin checks which occur every 5-years. • **No overdiagnosis** incorporated. • **No validation** of the model was performed.
Okafor 2013 [41]	• Model based on people with a diagnosis of Crohn’s Disease at 30 years of age and takes into account severity and remission states. • Age at Crohn’s disease diagnosis fixed at undefined distribution “around” 30 years. • Simulation of melanoma as proportions.	• **Varied diagnostic accuracy**. Sensitivity and specificity, false negatives resulting in later diagnosis • **Varied risk during lifetime** with subsequent NMSC diagnosis, age groups (< 50 and 50+) and exposure to thiopurines	• Age at keratinocyte cancer diagnosis not incorporated. • No information on time to next diagnosis or whether this varies by age at first diagnosis. **Overdiagnosis not** incorporated. • **No validation** of the model was performed. • **Sensitivity analyses** limited to one- and three-way • **No differentiation between in situ/low risk/high risk SCC or BCC.** Assumed localized disease requiring excision or metastatic disease. Survival differences by stage not incorporated into the simulation. • **Non-surgical options not considered** even with six-monthly and annual screening intervals
Walpole 2021 [52]	• Genetic status and test allocated at birth. Assume perfect compliance and test accuracy. • Surveillance starts at age 11, no variations in surveillance start time. • **No melanoma in situ** diagnoses. Given high-risk populations targeted this is unlikely to impact the intervention as the assumption that most in situ diagnoses would progress to invasive within an individuals lifetime is reasonable.	• Includes **distinct melanoma types** (uveal and cutaneous melanoma). • **Multiple lifetime melanoma events** (no simultaneous diagnoses). Once ‘cured’ of melanoma (alive after 10-years) subsequent events possible. • **Sensitivity analyses** for diagnostic rates and costs.	• Mutually exclusive uveal vs. cutaneous development. No individual develops both: this is inconsistent with the observed impact of BAP1 mutations on melanoma [84]. • Melanoma development and stage are **age independent**. This is inconsistent with the epidemiological characteristics of melanoma [84]. • **Restricted stages**: early vs. late diagnosis only. Probability of late diagnosis taken directly from observed healthcare presentation. • Assume **complete screening compliance**. No variations in uptake or adherence incorporated. Likelihood of **detection by doctor does not differ** by **any variables.** Assumes there is no delay between melanoma development and diagnosis. • No second event within 10 years. This is inconsistent with observed melanoma biology: ISPOR guideline II [84].
Wilson 2018 [47]	• No in situ melanoma state for nodular melanoma.	• Includes four distinct histological stubtypes (lentigo maligna, superficial spreading, acral lentiginous, and nodular melanoma). • Melanoma development and stage are age and sex dependent. • Explicitly simulates stage progression between all the AJCC stages [95], with stage-specific survival. • **Varied diagnostic accuracy**. Diagnoses could be false negatives and are dependent upon practitioner sensitivity.	• **Undiagnosed melanoma rates taken directly from incidence**. Undiagnosed prevalence based on a Northern Germany screening study. • Progression between stages is assumed to be histological subtype independent. However, progression from in situ to invasive depends upon histological subtype. • Assumes all melanoma encounters with clinicians are patient self-referred. This excludes the potential for a clinician to incidentally identify a melanoma in a clinical setting. • Single lifetime melanoma event. No recurrence or subsequent primaries are included. • Patients are assumed to have perfect adherence to guidelines. • No screening-related harms are included. • Treatment estimates are based on 2010 guidelines, which do not include immunotherapies.
Gogebakan 2020 [32]	• Screening only in high-risk individuals (self-assessed risk). The high-risk category was allocated as 20% of all melanoma cases. • Included only BRAF-positive persons and treatment efficacy. • No further treatment (post-excision) for early-stage patients. • Assume complete access to historic treatments and variable access to novel treatments. • Includes adjuvant treatment options for advanced melanoma	• **Separate simulations by sex.** • **Overdiagnosis estimates** incorporated. Implemented as an excess-incidence scheme. • **Survival dependent** upon treatment availability (period-based) and restricted stage groups. Stage-specific survival • **Sensitivity analyses** for overdiagnosis, stage-shift, size of risk group, hazard ratios of novel treatments. • Assume complete access to historic treatments and variable access to novel treatments.	• **No melanoma in situ**,** stage progression**,** or undiagnosed melanoma**. • Stage allocated at diagnosis • High-risk allocated equally between sexes. Risk scores include ‘ male’ as a risk factor: this assumption does not reflect knowledge about melanoma epidemiology [84]). • **All diagnoses accurate**. **No adverse events**. This underestimates the harms and costs associated with screening. • **Restricted stage** as early (Stage I-II) and advanced Stage III-IV) allocated at diagnosis. • Assume no further treatment (post-excision) for early-stage patients (or surveillance) • **Explicit stage shift** implemented from intervention (50% shift to early stage). • No within stage variation in survival. Survival variation between treatment implemented as hazard ratios. • **No self-detection**. Likelihood of **detection by doctor does not differ** by stage at diagnosis. Overdiagnosis captured as an **explicit excess in early diagnoses** where survival is 100**%**. • **No validation** of the model was performed.
Baltus 2025 [51] (based on Eisemann)	• People can only participate in the screening program until they have received a skin cancer diagnosis, and every person can get any of the three skin cancer entities only once. • Primary screening is performed at the general practitioner’s practice (58%) or dermatologist (42%). Sensitivity and specificity of skin cancer screening for dermatologists (91.7%, 81.5%) and GPs, (85%,75%) respectively. • Once a person has skin cancer diagnosed, they cannot re-enter the screening program this includes a BCC or SCC.	• Includes probability of diagnosis by stage at diagnosis for Melanoma and SCC and (in-situ Melanoma and SCC) for men and women. BCC is simulated un-staged (too much missing data). • Incorporates variation in screening capability between GP diagnosis (sensitivity 85%, specificity 75%) and dermatologist diagnosis (sensitivity 91.7%, specificity 81.5). **This was updated from Eisemann (2015).** • MM calculated estimates calibrated against national registry-derived stage. For BCC and SCC regional registry-derived data of observed age-standardised incidence for 2007 was used. 2007 was assumed to be a ‘pre-screening year’. **This was updated from Eisemann’s (2015) assumption.** • Validation of simulated outputs is discussed. • Sensitivity analyses for tumour growth rates and screening intervals. Variability assessed in terms of both input distributions and draws.	• The model does not incorporate any effects of patient self-selection or opportunistic screening. • Assumes constant risk. • Sensitivity and specificity for detection of skin cancers does not differentiate by skin cancer type. • Over- estimates deaths due to SCC. • Costs of follow-up and treatment were only included for the first-year post-diagnosis. No distinction in this time is made for skin cancers at different stages at diagnosis.
***Surveillance and management options***			
Claeson 2016 [48]	• Swedish population will grow by 8% up to 2023. • The Breslow thickness at diagnosis depends on the rate of growth and the time of development of the melanoma • 80% of patients with a fast-growing melanoma sought medical care within 15 months vs. 31 months for slow-growing melanoma.	• **Varied tumour growth rates**. Two growth rates simulated to mimic fast vs. slow developing melanomas. • **Diagnostic rates depend** on tumour thickness. • Model parameters **validated** against historical hospital data and literature.	• No clear model structure. Calibration and validation was performed but the process was unclear [84]. • **No melanoma in situ** diagnoses. • Melanoma rates are **age independent**. This is inconsistent with the epidemiological characteristics of melanoma [84]. No demographic or risk changes can be considered. • Explicitly **fixed yearly increase** (5.25%) in total melanoma rates over time. This means no primary prevention trends can be incorporated. • **Fixed delay** to clinician attendance (1 month). This does not stratify for site or patient characteristics which may change attendance patterns. • **All diagnoses accurate**. **No self-detection included and l**ikelihood of **detection by doctor does not differ** by tumour growth rates.
Kontogiannis 2022 [44]	• Modelled patients with stage IA and IB melanoma separately. • Staged recurrence - probability of recurrence, metastases and occurrence of a new primary in a monthly cycle. • Assumes entrance in full health (disease free) after melanoma excision. • Excluded strategies including risk models, diagnostic imaging, and GP surveillance due to lack of evidence. Three surveillance variables: clinical specialty (dermatologist, surgeon or melanoma nurse), surveillance intervals, duration of follow-up. • Assume Stage IA disease does not change mortality, no impact on overall survival. • Cycle length 1 month. • Different follow-up durations Stage I 10 years and Stage II 20 years. • Recurrence probabilities based on Australian data	• Diagnosed by patients or person with experience in diagnosing melanoma. Includes false positives and false negative diagnoses. Includes undiagnosed progression of melanoma. • **Varied diagnostic accuracy** based on level of practitioner expertise and pathologist staging of melanoma. • **Survival dependent** upon stage at diagnosis, age, and sex. • PSAs run for self-detection, recurrence prob, utility scores, clinician accuracy. EVPPI analysis on utilities, diagnostic accuracy, prob progression and prob recurrence. One-way sensitivity analyses • Self-detection and seeking treatment for recurrence and new primary. Included costs for over-anxious screening “false alarm”.	• **No melanoma in situ** diagnoses. • Mixed demographic inputs (UK, German, Australian, and Dutch). This increases potential parameter space. • Clinical accuracy based on Australian rates (reportedly distinct from UK). • Second primaries assumed to be stage 1. Utilities assumed the same for first and subsequent diagnosis. • Recurrence rates for Stage III and IV melanomas were assumed to be the same as stage IIC. • **No validation** of the model was performed. Small simulation runs. • Probability of recurrence or a new primary were the same but varied based on AJCC stage
van der Meijde 2016 [49]	• Tumour growth as per rate limiting steps theory. • Separate calibration steps for tumour and survival parameters (using German targets, Dutch validation). • Dwelling times in observable growth states only decrease with size (up to a symptomatic largest size tumour where individual remains until diagnosis). • No local recurrence possible after stage III treatment. • Includes treatment options • Treatment changes tumour starting states: if treatment “cures” no subsequent melanoma growth is possible.	• Explicitly simulated tumour growth and patient level components. • Tumour states influenced by treatment module. • Symptomatic state influenced by tumour size and location. Recurrence can be local, regional or distant (simulated in parallel), accounts for unobservable tumour presence. • Includes **variation in surveillance accuracy**. Non-surveillance-based detection is possible from the symptomatic state. • **Calibrated** values for unobservable states with multiple targets. Multiple calibration runs. **Validated** against Dutch stage-specific overall survival. No incidence validation against Dutch data. • Variation in test positivity between tests (and based on surveillance or diagnosis) depends upon tumour state, site, and patient features. • Clinical features simulated separately: allows assessment of changing multiple interventions simultaneously. • Can assign molecular characteristics to tumour chains: can simulate molecular diagnostic techniques and targeted treatments. • Tumour states influenced by treatment module. • Allows simulation of treatment-resistant developments.	• **No melanoma in situ** diagnoses. • No undiagnosed melanoma/well states. • No local recurrence possible after stage III treatment. • Local or regional recurrence cannot result in melanoma specific death. This is inconsistent with observed melanoma biology [84]. • German data used to inform progression and recurrence parameters. • Stage IV disease calibrated against survival from 1996–2010: excludes improved survival due to immunotherapies. • Single treatment phase for metastatic disease (time-to-death only), doesn’t simulate alternative adjuvant therapy options.
***Diagnosis option***			
van der Meijde 2016 [49]	• Patients entered the Markov model at Stage I-IV based on melanoma status based on diagnostic accuracy • Microscopic assessment conducted by physicians with various levels of expertise rather than pathologist	• Incorporates ‘detection and miss rates’ related to melanoma diagnosis • Included overdiagnosis costs and utilities.	• No clear description of Markov model structure. • Calibration and validation of model was not reported • **No melanoma in situ** diagnoses. • Disease progression or recurrence risk related to stage of disease not explained • Survival based on SEER data • No justification for ranges of up to 25% in sensitivity analysis
***Treatment options***			
Almutairi 2019 [39]	• Based on phase2 data and patients with metastatic disease remain progression free or move to progression or death • Progression between the states occurs following either a transition to death or to a more advanced stage. Separate transition probabilities apply.	• Adverse events modelled Sensitivity estimates included surrounding simulation error only.	• Assume treatment effects (quality of life) are the same for all patients • No separation between progression and death; counted as one state within the simulation • Assumes treatments had the same efficacy and response between patient subgroups. • How withdrawals handled not described • No validation reported • Only simulated a 100 patient cohort, sensitivity analyses included 2000 simulation runs.
Beale 2013 [45]	• Reassessment of model used for Health technology assessment and application for public funding for new adjuvant therapy.	• Adverse events modelled	• Effects of cross-over of patients in trial not adjusted for. • Assume treatment effects (quality of life) are the same for all patients • Assumes treatments had the same efficacy and response between patients. • Long-term survival between 45 months and 10 years based on a single mortality risk parameter • No validation reported
Crott 2004 [42]	• Based on trial participant demographics and data (7 years) and extrapolated to lifetime (35 years) • Long-term recurrence patients do not survive beyond 12 months. Time from Rx to Death independent of time between diagnosis and treatment.	• Varied recurrence/death probabilities. • Varied dose compliance (60,80,100%), varied utility estimates • **Sensitivity analyses** for post-trial death rates, estimated utility of treatment.	• Disease specific survival only, no competing OC mortality. • Toxicities only possible in first year. Toxicities taken from E1684 only: high-dose Tx only. • No difference between local/systemic recurrence (outcomes weighted towards distant metastases). • No difference in follow-up intensity between arms. • No validation of extrapolation [84]. • Long term time horizon of 35 years may be unrealistic for stage III in 2014. • After year 7, annual constant recurrence probability which was halved at year 10.
Gibson 2019 [43]	• Patient level simulation was one of multiple models compared. Events were set to those in non-patient-level models. • Paired patient simulations. • To allow comparison to Markov models, baseline patient characteristics were the average of the trial patients. Heterogeneity only included as scenario.	• Survival independently incorporated modelled for each treatment. Different survival distributions implemented based on trial end points (prog free survival and overall survival)	• No adverse events modelled. • No explicit tumour growth. Model fit to survival based on visual inspection and goodness-of-fit criteria. [Note] Most limitations are a result of deliberate model design for comparison with Markov models.
Li 2023[34]	• First intervention varies, all individuals receive same second-line treatment, followed by best-supportive care. • Average weight used to adjust treatment costs. Did not include possible adjuvant treatments for second-line therapy.	• **Sensitivity analyses** for all model parameters. • Adverse events included. Distinct survival based on possible treatments available (some distribution selection). • Includes treatment discontinuation choice (for second-line treatment only). • Incorporates survival estimates on the basis of known first-line treatments.	• No undiagnosed melanoma/well states. • No variation in melanoma states (by stage or histological subtype). • No melanoma-specific death (adverse event death only). • Fixed transition probabilities (survival/PFS is age independent)
Mojtahed 2021 [36]	• Single start age and weight. • Assume treatment discontinued after AE (not always clinically accurate). • 2-years RFS deemed cured (not further death from melanoma possible, background mortality rate). • Local recurrence has two treatment options (not adjuvant), distant recurrence means second-line treatment (100% uptake). 50/50 second-line adjuvant if first-line adjuvant refused. • Single-point (18month) back-calculated survival. All treatments only for one year.	• **Sensitivity analyses** for all model parameters and for 2-year cure (up to 5-year cure). PSAs for 1000 simulations. • Includes survival rates and adverse events rates. AE and recurrence cannot occur at the same time. No adverse events possible after distant metastasis.	• No undiagnosed melanoma/well states. • Transition probability are time independent (treatment impact can vary over time). • Survival does not take into account progression (set from initial state or recurrent state). Assume complete resection.
Seidler 2009 [37]	• Single oncologist assessment of margins/repair type. All deep margins were assumed to be clear. • Grouped BCCs and SCCs.	• Site, size and subtype impact outcomes. • **Sensitivity analyses** for all model parameters. PSA for costs, recurrence probabilities, and utilities.	• Fixed recurrence rate (1% or 5% over 5-years, intervention dependent). • No survival variation. No survival impacts.
Tarhini 2018 [38]	• Patient characteristics include age, sex, race, weight, and smoking. • Explicit simulation of treatment start and end, with potential for treatment discontinuation. • Time since diagnosis only incorporated into 1 treatment line.	• Progression possible during and after treatment. • Adverse costs/utilities included (no survival impacts of adverse events), only grade 3and4 adverse events incorporated. • Number of lesions changes treatment response. • **Sensitivity analyses** for all model parameters. PSA for 1000 simulations.	• Excel implementation is inappropriate [84] • Internal validation only (compared with trials used to parameterise). • Disease stage not included in risk equations (which change transitions). Metastasis stage do influence transitions.
Lee 2019 [46]	• Model inputs for both models based on same trial data excluding survival data variables. • Patient level simulation was one of two models compared to examine differences in outcome if survival data not available.	• **Adverse events** considered. • **Sensitivity analyses** for all model parameters. PSA for 1000 simulations. • **Checked extended survival estimates against expert opinion.**	• No explicit simulation of disease stages; disease either progression-free or progressed, There is no survival variation based on stage. Patient population characteristics and stage at entry not described. • Limited survival data. • Assumed treatments had same efficacy, response and treatment related effects (quality of life) for all patients. • How withdrawals from treatment were handled was not described.

AI, artificial intelligence; CD Crohn’s disease; MSM, melanoma-specific mortality; NMSC non melanoma skin cancer, OCM, other-cause mortality, SEER Surveillance, Epidemiology, and End Results program, PSA Probabilistic sensitivity analysis, BCC basal cell carcinoma; SCC Squamous cell carcinoma; AJCC The American Joint Committee on Cancer;

^1^ As referred to in the ISPOR guidelines or mentioned specifically in the Table [84]

^2^ Model characteristics described related to details related to the development of melanoma simulation model within the Australian population

Only assessments of the model characteristics are performed (no assessment of the costs or utility aspects to the papers). The models are separated by the aspect of melanoma life history they were developed to simulate; (1) before melanoma development to allow assessment of preventative interventions, (2) natural history of melanoma to assess screening and early detection methods, (3) post-diagnosis management for surveillance assessments, and (4) clinical pathways to assess treatment options. The strengths and limitations include those identified by the original model authors with additional comments from these authors. Explanations of the allocation of assumptions to strengths or limitations are added (based on the ISPOR guidelines [84]), with common assumptions highlighted in bold. Where ISPOR guideline II is cited, there has been no attempt to explain the reasoning behind the assumptiions

All models incorporated mortality risk. This was usually based on Breslow thickness of the melanoma at diagnosis [35, 47, 48, 50] using registry or trial data or by simulating melanoma growth rates [47–51].

### Quality assessment

There was heterogeneity in the structure and complexity of the models in this review underpinned by variations in the key events and outcomes of interest. The checklist with the quality assessment scores of reviewed studies is provided in Supplementary Tables 1 and further details on the strengths, limitations, and assumptions of the models related to their applicability for skin cancer screening is shown in Table 2. Based on the QAAMS checklist, five models were considered high quality [43, 45, 46, 49, 51] and the remaining models were assessed as moderate quality. Generally, lower scores were related to validity and scope of results, not describing whether calibration or internal and external validation were conducted or the methods applied, and limited discussion around generalisability.

All papers clearly described the purpose of the model and target populations and all but one [48] of the models clearly described the model structures. The model parameter estimations and the data sources used to inform both the model design and parameterisation were generally well described. Validation, if conducted, mostly used population or patient data. The reporting of results included sensitivity analyses in all studies. Uncertainty due to data limitations was often noted, such as for the natural history of skin cancer, the melanoma growth rate in an undiagnosed state, time to diagnosis, and diagnostic accuracy. For melanoma growth rates, estimations were often based on other models or expert guidance. Not all models incorporated variation in diagnostic accuracy with respect to the mode of diagnosis. The availability of models and technical documentation was noted in some papers. Three papers provided information regarding reproducible research [32, 47, 49].

## Discussion

To our knowledge, this is the first review of skin cancer skin cancer microsimulation models. The majority of the models focused on late-stage melanoma treatment strategies, whereas fewer models evaluated skin cancer screening. We identified many limitations of existing models, although it is important to recognise that each model was designed with a specific purpose in mind and that the limitations may only apply to certain applications of the model. Assessing the structure, parameterisation, and assumptions of skin cancer microsimulation models will inform the development of new skin cancer models as well as help to improve existing models, including those related to skin cancer treatments. Similar to a systematic review of simulation studies of cancer screening [17], we also found a trend toward increasing quality of models over time.

Microsimulation models have been mostly used to address gaps in evidence related to the comparative efficacy of melanoma treatment strategies tested in clinical trials, and thus most have focused on survival outcomes within small populations. We also identified some knowledge gaps related to modelling time to diagnosis where the models commenced following the diagnosis of a melanoma or keratinocyte cancer, avoiding the preclinical or undiagnosed state. Only three models included either detection or management of keratinocyte cancer. No models included melanoma and keratinocyte cancers together despite their common co-occurrence [2, 59] and impact on healthcare costs [14].

In Australia, a substantial proportion of melanomas are initially detected by patients, who present to a doctor with a lesion of concern [11]. Thus, models assessing screening should incorporate skin checks that are patient-driven or occurring opportunistically alongside an organised screening program, as this reflects the real-world setting. While none of the simulations distinguished melanoma diagnosed at routine skin checks from those detected by a doctor opportunistically or incidentally, this factor should be considered in countries where routine skin checks are common such as Australia [11, 60]. Another behavioural factor to be considered in Australia is related to geographical remoteness. People living in rural and remote areas comprise approximately 28% of the population and have higher levels of preventable illness and avoidable hospitalisations compared to people living in metropolitan areas [61]. One of the reasons for this disparity is the limited availability of health services as the population is more dispersed [61].Simulation models should take into account behavioural characteristics [62] that can impact cost-effectiveness and inform broader policy considerations [63].

In addition, the ratio of in situ to invasive melanoma diagnoses has been increasing over time in several countries [64, 65], and in Queensland two thirds of melanomas are diagnosed at the in situ stage (stage 0) [66]. While the risk of a new melanoma and the mean age of diagnosis of in situ melanomas is similar to invasive melanoma [66, 67], in situ melanomas do not generally cause death. However, they have similar clinical management, excision costs, and psychological impacts as early-stage invasive melanomas [68]. When assessing population screening programs, estimates of harms from screening need to be considered, including the risk of overdiagnosis [66] referring to diagnosis of melanomas and other skin cancers that were never going to cause harm if left undiagnosed and untreated [69].

Over half of in situ melanomas diagnosed are estimated to be over diagnosed, compared to 15% of invasive melanomas [70]. In our review, two models included in situ melanoma [47, 51] and one model included risk of subsequent melanoma [52]. Future models should ideally incorporate in situ melanomas, melanoma risk factors [6] and subsequent primary melanoma [71], because individuals with a previous melanoma are at substantially increased risk of developing another primary melanoma. Other lesions that are identified should also be accounted for as other procedures will incur additional costs and may lead to harms such as removal of benign lesions, scarring and psychological impacts [72].

None of the models in this review incorporated variations in ethnicity or skin colour, but as models become more sophisticated, heterogeneity of the population should be included to reflect population diversity [73]. Three models included cohorts of a given age, sex and risk score [32, 47, 51], but no models took into account risk factor trends over time such as period or birth-cohort effects as a result of melanoma and sun protection awareness campaigns [74, 75], which are associated with decreasing melanoma incidence rates in younger age groups [76, 77].

There are several limitations of our review. Because we only reviewed microsimulation models and did not include cohort models or partitioned models, our findings may not be generalisable to all health economic models that have examined the costs and benefits of skin cancer screening. Data limitations were noted in several papers. Ideally, locally representative data are preferable for model development. One model for the UK population [44] used Australian data to inform parameter selection. As Australia has higher melanoma rates [78], this may result in over-estimation of melanoma incidence and recurrence rates within the simulation. As noted with microsimulation models of colorectal cancer [79], the challenges in modelling progression from a lesion to a cancer has led to a range of assumptions in different models. We also note that all models in the systematic review were in English and hence we may have missed some differently indexed publications, and we did not consider the grey literature.

In a recently developed model by Wilson et al. [80], incorporation of preclinical non-detectable and detectable states varied by melanoma subtype and influenced diagnosis. However, once melanoma was considered invasive, it was assumed there was no difference in the rate of progression of disease by subtype. When estimating growth rate of skin cancers, Bathus et al. considered tumour thickness, but not melanoma subtype [51]. In this review, most models assumed an average tumour behaviour, which is a limitation given that there are known distinct clinical and biological differences between melanoma subtypes that influence growth rates and prognosis [81]. Linking tumour growth rates with potential screening disruption to the tumour’s natural history is necessary to accurately estimate the potential lives saved due to skin cancer screening. Identified gaps in this review were around modelling of natural history and disease progression of skin cancer, and incorporating behavioural aspects such as patient self-selection or opportunistic screening.

Microsimulation models are uniquely positioned to simulate different scenarios and develop approaches to address individual-level variation under a variety of settings. The value of models for assessing potential screening programs is in being able to generate a range of simulated screening scenarios, facilitating the comparison of the relative benefits, harms and costs of a range of strategies before the implementation of a screening policy in the real world. Our review identified relatively few microsimulation models of skin cancer screening and identified common limitations that can be addressed in the development of Policy-1 Melanoma, a comprehensive microsimulation model of skin cancer screening.

## Electronic supplementary material

Below is the link to the electronic supplementary material.

Supplementary Material 1

## Data Availability

The datasets used and/or analysed during the current study are available from the corresponding author on reasonable request.
