# Supplementary material for: A systematic review of microsimulation models for skin cancer
Source: BMC Med Inform Decis Mak. 2025 Jul 1;25:222. doi: 10.1186/s12911-025-03074-9 (PMC12211653; doi:10.1186/s12911-025-03074-9)
Supplement: Supplementary file 1 — Supplementary Material 1 [file 12911_2025_3074_MOESM1_ESM.docx]

**Supplementary Materials**

**Search strategy for systematic review (to May 7 2025)**

1 melanoma.mp. or Melanoma/

2 limit 1 to yr="1987 -Current"

3 skin cancer.mp.

4 limit 3 to yr="1987- Current"

5 microsimulation.mp.

6 limit 5 to yr="1987- Current"

7 microsim*.mp.

8 agent-based model*.mp.

9 limit 8 to yr="1987- Current"

10 microsim*.mp.

11 limit 10 to yr="1987- Current"

12 individual-based model*.mp.

13 individual-based model*.mp. .

14 limit 13 to yr="1987- Current"

15 individual-based model.mp.

16 9 or 11 or 14

17 2 or 4

18 16 and 17

Further details at https://www.crd.york.ac.uk/PROSPERO/view/CRD42024504250

**Policy-1 modelling platform**

The Policy1 suite of modelling platforms have been developed over the past two decades by researchers at the University of Sydney and the Daffodil Centre (a joint venture between Cancer Council NSW and the University of Sydney), with support from Australian and international public health experts and clinicians. The platform has informed policymakers in Australia(1-3), New Zealand(4, 5), the United Kingdom(6), and the United States(7, 8). Notably, Policy1-Cervix has played a critical role in the development of cervical cancer screening guidelines by the World Health Organization (WHO)(9, 10) and was a key member of the modelling consortium that supported the development of the WHO Global Strategy for cervical cancer elimination(11, 12).

Development and refinement of the Policy1 models have been supported by funding from several Australian agencies, including the National Health and Medical Research Council (NHMRC) and the Medical Research Future Fund (MRFF), as well as international research organizations such as the National Institutes of Health (NIH), Cancer Research UK and WHO. Building on this established expertise, Policy1-Melanoma aims to assess the cost-effectiveness, benefits, and potential harms of risk-based melanoma screening. Furthermore, this model will undergo continuous development to serve as a robust and adaptable infrastructure for evaluating emerging technologies and novel screening strategies for melanoma.

**Supplementary Table 1**. Quality Assessment Reporting for Microsimulation Models (QARMM) checklist(36)

|  | Almutairi (13) | Beale(14) | Claeson(15) | Crott(16) | Eisemann(17) | Eskander(18) | Gibson(19) | Gogebakan (20) | Gomez Rossi (21) | Kansal(22) | Kontogiannis(23) | Lee (24) | Li (25) | Losina(26) | Mojtahed (27) | Okafor (28) | Romero(29) | Seidler(30) | Tarhini(31) | *van der Meijde(32)* | Walpole(33) | Wilson(34) | Baltus(35) |
| --- | --- | --- | --- | --- | --- | --- | --- | --- | --- | --- | --- | --- | --- | --- | --- | --- | --- | --- | --- | --- | --- | --- | --- |
| Are the objectives/goals of the model well defined? | 1 | 1 | 1 | 1 | 1 | 1 | 1 | 1 | 1 | 1 | 1 | 1 | 1 | 1 | 1 | 1 | 1 | 1 | 1 | 1 | 1 | 1 | 1 |
| Is the target population described appropriately? | 0 | 0 | 0.5 | 1 | 0.5 | 1 | 0.5 | 0 | 0.5 | 1 | 0.5 | 0 | 1 | 1 | 1 | 1 | 0.5 | 0 | 1 | 0 | 1 | 1 | 1 |
| Are the data used in the model development representative of the population examined? | 0.5 | 1 | 1 | 1 | 1 | 1 | 1 | 1 | 1 | 1 | 1 | 0.5 | 1 | 1 | 1 | 1 | 0.5 | 0 | 0 | 1 | 0.5 | 1 | 1 |
| Are the data sources informing parameter estimations provided | 0.5 | 0.5 | 0.5 | 0 | 0 | 1 | 1 | 0.5 | 1 | 0.5 | 1 | 1 | 1 | 1 | 1 | 1 | 0.5 | 0 | 0 | 1 | 1 | 0.5 | 1 |
| Are the parameters used to populate model frameworks specified? | 0.5 | 0.5 | 0.5 | 0 | 1 | 1 | 1 | 1 | 0.5 | 0.5 | 1 | 1 | 1 | 1 | 1 | 1 | 0.5 | 1 | 0 | 1 | 1 | 1 | 0.5 |
| Is the model structure well described (e.g., are assumptions clear, are there choices for the user to make? | 1 | 0.5 | 0.5 | 0.5 | 0.5 | 1 | 0.5 | 1 | 1 | 1 | 1 | 1 | 1 | 1 | 1 | 1 | 1 | 1 | 1 | 1 | 0.5 | 1 | 0.5 |
| Is the time horizon of the model provided? | 1 | 1 | 1 | 1 | 1 | 1 | 1 | 0 | 1 | 1 | 1 | 0.5 | 1 | 0.5 | 1 | 1 | 1 | 1 | 0.5 | 0.5 | 1 | 1 | 1 |
| Are all simulated strategies/scenarios specified and/or explained clearly? | 0.5 | 1 | 0.5 | 1 | 1 | 1 | 1 | 1 | 0.5 | 1 | 1 | 1 | 0.5 | 0.5 | 0.5 | 1 | 1 | 1 | 0 | 1 | 0.5 | 1 | 1 |
| Are model uncertainties discussed? | 1 | 1 | 0.5 | 0.5 | 0.5 | 0.5 | 0.5 | 0.5 | 0.5 | 0.5 | 1 | 1 | 0 | 0.5 | 0 | 0.5 | 0 | 0 | 1 | 1 | 0.5 | 1 | 1 |
| Are sensitivity analyses performed and reported? | 1 | 1 | 0 | 1 | 1 | 1 | 1 | 1 | 1 | 1 | 1 | 1 | 1 | 1 | 1 | 1 | 0.5 | 0 | 1 | 0 | 1 | 0 | 1 |
| Is model calibration performed and reported, where required? | 0 | 1 | 0.5 | 0 | 1 | 0 | 1 | 0 | 0 | 0 | 0 | 0 | 0 | 0 | 0.5 | 0 | 0 | 0 | 0 | 1 | 0 | 0 | 1 |
| Is internal and/or external validation performed and reported? | 0 | 0 | 0.5 | 0 | 0.5 | 0 | 0.5 | 0 | 0 | 0 | 0.5 | 1 | 1 | 0 | 0 | 0 | 1 | 1 | 1 | 1 | 0 | 0.5 | 1 |
| Is predictive validation performed and/or attempted? | 0 | 1 | 0 | 0 | 0 | 0 | 1 | 0 | 0 | 0 | 0 | 1 | 0 | 0 | 0 | 0 | 0 | 0 | 0 | 1 | 0 | 0 | 0.5 |
|  | Almutairi (13) | Beale(14) | Claeson(15) | Eiseman | Eisemann(17) | Eskander(18) | Gibson(19) | Gogebakan (20) | Gomez Rossi (21) | Kansal(22) | Kontogiannis(23) | Lee (24) | Li (25) | Losina(26) | Mojtahed (27) | Okafor (28) | Romero(29) | Seidler(30) | Tarhini(31) | *Van der Meijde(32)* | Walpole(33) | Wilson(34) | Baltus(35) |
| Is model generalisability discussed? | 1 | 1 | 0 | 0 | 1 | 0 | 0 | 0 | 0 | 0 | 0 | 1 | 0.5 | 0 | 0.5 | 0 | 0.5 | 0 | 1 | 1 | 0.5 | 0 | 1 |
| Are model limitations discussed? | 1 | 1 | 1 | 0 | 1 | 0 | 0.5 | 1 | 1 | 0.5 | 1 | 0.5 | 1 | 1 | 1 | 0 | 0 | 0.5 | 1 | 1 | 0.5 | 1 | 0.5 |
| Score | 9 | 11.5 | 8 | 7 | 11 | 9.5 | 11.5 | 8 | 9 | 9 | 11 | 11.5 | 11 | 9.5 | 10.5 | 9.5 | 8 | 6.5 | 8.5 | 12.5 | 9 | 10 | 13 |

**Supplementary Table 1**. Quality Assessment Reporting for Microsimulation Models (QARMM) checklist(36)

**References**

1. Hall MT, Simms KT, Lew J-B, Smith MA, Brotherton JML, Saville M, et al. The projected timeframe until cervical cancer elimination in Australia: a modelling study. The Lancet Public Health. 2019;4(1):e19-e27.

2. Lew J-B, St John DJB, Xu X-M, Greuter MJE, Caruana M, Cenin DR, et al. Long-term evaluation of benefits, harms, and cost-effectiveness of the National Bowel Cancer Screening Program in Australia: a modelling study. The Lancet Public Health. 2017;2(7):e331-e40.

3. Behar Harpaz S, Weber MF, Wade S, Ngo PJ, Vaneckova P, Sarich PEA, et al. Updated cost-effectiveness analysis of lung cancer screening for Australia, capturing differences in the health economic impact of NELSON and NLST outcomes. British Journal of Cancer. 2023;128(1):91-101.

4. Smith MA, Hall M, Lew J-B, Canfell K. Potential for HPV vaccination and primary HPV screening to reduce cervical cancer disparities: Example from New Zealand. Vaccine. 2018;36(42):6314-24.

5. Hall MT, Smith MA, Lew J-B, O'Hallahan J, Fentiman G, Neal H, et al. The combined impact of implementing HPV immunisation and primary HPV screening in New Zealand: Transitional and long-term benefits, costs and resource utilisation implications. Gynecologic Oncology. 2019;152(3):472-9.

6. Simms KT, Smith MA, Lew J-B, Kitchener HC, Castle PE, Canfell K. Will cervical screening remain cost-effective in women offered the next generation nonavalent HPV vaccine? Results for four developed countries. International Journal of Cancer. 2016;139(12):2771-80.

7. Burger EA, de Kok IMCM, O'Mahony JF, Rebolj M, Jansen EEL, de Bondt DD, et al. A model-based analysis of the health impacts of COVID-19 disruptions to primary cervical screening by time since last screen for current and future disruptions. eLife. 2022;11:e81711.

8. Burger EA, Jansen EEL, de Bondt D, Killen J, Spencer JC, Regan MC, et al. Disparities in cervical cancer elimination time frames in the United States: a comparative modeling study. JNCI: Journal of the National Cancer Institute. 2025.

9. Simms KT, Keane A, Nguyen DTN, Caruana M, Hall MT, Lui G, et al. Benefits, harms and cost-effectiveness of cervical screening, triage and treatment strategies for women in the general population. Nature Medicine. 2023;29(12):3050-8.

10. Hall MT, Simms KT, Murray JM, Keane A, Nguyen DTN, Caruana M, et al. Benefits and harms of cervical screening, triage and treatment strategies in women living with HIV. Nature Medicine. 2023;29(12):3059-66.

11. Canfell K, Kim JJ, Brisson M, Keane A, Simms KT, Caruana M, et al. Mortality impact of achieving WHO cervical cancer elimination targets: a comparative modelling analysis in 78 low-income and lower-middle-income countries. The Lancet. 2020;395(10224):591-603.

12. Brisson M, Kim JJ, Canfell K, Drolet M, Gingras G, Burger EA, et al. Impact of HPV vaccination and cervical screening on cervical cancer elimination: a comparative modelling analysis in 78 low-income and lower-middle-income countries. The Lancet. 2020;395(10224):575-90.

13. Almutairi AR, Alkhatib NS, Oh M, Curiel-Lewandrowski C, Babiker HM, Cranmer LD, et al. Economic Evaluation of Talimogene Laherparepvec Plus Ipilimumab Combination Therapy vs Ipilimumab Monotherapy in Patients With Advanced Unresectable Melanoma. JAMA Dermatol. 2019;155(1):22-8.

14. Beale S, Dickson R, Bagust A, Blundell M, Dundar Y, Boland A, et al. Vemurafenib for the treatment of locally advanced or metastatic BRAF V600 mutation-positive malignant melanoma: a NICE single technology appraisal. Pharmacoeconomics. 2013;31(12):1121-9.

15. Claeson M, Hallberg S, Holmstrom P, Wennberg AM, Gonzalez H, Paoli J. Modelling the Future: System Dynamics in the Cutaneous Malignant Melanoma Care Pathway. Acta Derm Venereol. 2016;96(2):181-5.

16. Crott R, Ali F, Burdette-Radoux S. Cost-utility of adjuvant high-dose interferon alpha therapy in stage III cutaneous melanoma in Quebec. Value Health. 2004;7(4):423-32.

17. Eisemann N, Waldmann A, Garbe C, Katalinic A. Development of a microsimulation of melanoma mortality for evaluating the effectiveness of population-based skin cancer screening. Med Decis Making. 2015;35(2):243-54.

18. Eskander A, Marqueen KE, Edwards HA, Joshua AM, Petrella TM, de Almeida JR, et al. To ban or not to ban tanning bed use for minors: A cost-effectiveness analysis from multiple US perspectives for invasive melanoma. Cancer. 2021;127(13):2333-41.

19. Gibson EJ, Begum N, Koblbauer I, Dranitsaris G, Liew D, McEwan P, et al. Cohort versus patient level simulation for the economic evaluation of single versus combination immuno-oncology therapies in metastatic melanoma. J Med Econ. 2019;22(6):531-44.

20. Gogebakan KC, Berry EG, Geller AC, Sonmez K, Leachman SA, Etzioni R. Strategizing Screening for Melanoma in an Era of Novel Treatments: A Model-Based Approach. Cancer Epidemiol Biomarkers Prev. 2020;29(12):2599-607.

21. Gomez Rossi J, Rojas-Perilla N, Krois J, Schwendicke F. Cost-effectiveness of Artificial Intelligence as a Decision-Support System Applied to the Detection and Grading of Melanoma, Dental Caries, and Diabetic Retinopathy. JAMA Netw Open. 2022;5(3):e220269.

22. Kansal AR, Shaul AJ, Stern S, Busam K, Doucet CA, Chalfin DB. Cost-effectiveness of a FISH assay for the diagnosis of melanoma in the USA. Expert Rev Pharmacoecon Outcomes Res. 2013;13(3):371-80.

23. Kontogiannis V, Coughlan D, Javanbakht M, Kunonga P, Beyer F, Richmond C, et al. Optimal Surveillance Strategies for Early-Stage Cutaneous Melanoma Post Primary Tumor Excision: An Economic Evaluation. MDM Policy Pract. 2022;7(1):23814683211069988.

24. Lee D, Amadi A, Sabater J, Ellis J, Johnson H, Kotapati S, et al. Can We Accurately Predict Cost Effectiveness Without Access to Overall Survival Data? The Case Study of Nivolumab in Combination with Ipilimumab for the Treatment of Patients with Advanced Melanoma in England. Pharmacoecon Open. 2019;3(1):43-54.

25. Li SN, Wan X, Peng LB, Li YM, Li JH. Cost-effectiveness of immune checkpoint inhibition and targeted treatment in combination as adjuvant treatment of patient with BRAF-mutant advanced melanoma. BMC Health Serv Res. 2023;23(1):49.

26. Losina E, Walensky RP, Geller A, Beddingfield FC, 3rd, Wolf LL, Gilchrest BA, et al. Visual screening for malignant melanoma: a cost-effectiveness analysis. Arch Dermatol. 2007;143(1):21-8.

27. Mojtahed SA, Boyer NR, Rao SA, Gajewski TF, Tseng J, Turaga KK. Cost-Effectiveness Analysis of Adjuvant Therapy for BRAF-Mutant Resected Stage III Melanoma in Medicare Patients. Ann Surg Oncol. 2021;28(13):9039-47.

28. Okafor PN, Stallwood CG, Nguyen L, Sahni D, Wasan SK, Farraye FA, et al. Cost-effectiveness of nonmelanoma skin cancer screening in Crohn's disease patients. Inflamm Bowel Dis. 2013;19(13):2787-95.

29. Romero HL, Dellaert NP, van der Geer S, Frunt M, Jansen-Vullers MH, Krekels GA. Admission and capacity planning for the implementation of one-stop-shop in skin cancer treatment using simulation-based optimization. Health Care Manag Sci. 2013;16(1):75-86.

30. Seidler AM, Bramlette TB, Washington CV, Szeto H, Chen SC. Mohs versus traditional surgical excision for facial and auricular nonmelanoma skin cancer: an analysis of cost-effectiveness. Dermatol Surg. 2009;35(11):1776-87.

31. Tarhini A, Benedict A, McDermott D, Rao S, Ambavane A, Gupte-Singh K, et al. Sequential treatment approaches in the management of BRAF wild-type advanced melanoma: a cost-effectiveness analysis. Immunotherapy. 2018;10(14):1241-52.

32. van der Meijde E, van den Eertwegh AJ, Linn SC, Meijer GA, Fijneman RJ, Coupe VM. The Melanoma MAICare Framework: A Microsimulation Model for the Assessment of Individualized Cancer Care. Cancer Inform. 2016;15:115-27.

33. Walpole S, Hayward NK, Pritchard AL, Johansson PA. Microsimulation Model for Evaluating the Cost-Effectiveness of Surveillance in BAP1 Pathogenic Variant Carriers. JCO Clin Cancer Inform. 2021;5:143-54.

34. Wilson ECF, Usher-Smith JA, Emery J, Corrie P, Walter FM. A Modeling Study of the Cost-Effectiveness of a Risk-Stratified Surveillance Program for Melanoma in the United Kingdom. Value Health. 2018;21(6):658-68.

35. Baltus H, Hübner J, Garbe C, Hagenström K, Rohr M, Hischke S, et al. Evaluation of skin cancer screening in Germany - a Microsimulation. J Dtsch Dermatol Ges. 2025;23(1):19-27.

36. de Oliveira C, Matias MA, Jacobs R. Microsimulation Models on Mental Health: A Critical Review of the Literature. Value in Health. 2024;27(2):226-46.
